# Characterization of a new SARS-CoV-2 variant that emerged in Brazil

**DOI:** 10.1073/pnas.2106535118

**Published:** 2021-06-17

**Authors:** Masaki Imai, Peter J. Halfmann, Seiya Yamayoshi, Kiyoko Iwatsuki-Horimoto, Shiho Chiba, Tokiko Watanabe, Noriko Nakajima, Mutsumi Ito, Makoto Kuroda, Maki Kiso, Tadashi Maemura, Kenta Takahashi, Samantha Loeber, Masato Hatta, Michiko Koga, Hiroyuki Nagai, Shinya Yamamoto, Makoto Saito, Eisuke Adachi, Osamu Akasaka, Morio Nakamura, Ichiro Nakachi, Takayuki Ogura, Rie Baba, Kensuke Fujita, Junichi Ochi, Keiko Mitamura, Hideaki Kato, Hideaki Nakajima, Kazuma Yagi, Shin-ichiro Hattori, Kenji Maeda, Tetsuya Suzuki, Yusuke Miyazato, Riccardo Valdez, Carmen Gherasim, Yuri Furusawa, Moe Okuda, Michiko Ujie, Tiago J. S. Lopes, Atsuhiro Yasuhara, Hiroshi Ueki, Yuko Sakai-Tagawa, Amie J. Eisfeld, John J. Baczenas, David A. Baker, Shelby L. O’Connor, David H. O’Connor, Shuetsu Fukushi, Tsuguto Fujimoto, Yudai Kuroda, Aubree Gordon, Ken Maeda, Norio Ohmagari, Norio Sugaya, Hiroshi Yotsuyanagi, Hiroaki Mitsuya, Tadaki Suzuki, Yoshihiro Kawaoka

**Affiliations:** ^a^Division of Virology, Department of Microbiology and Immunology, Institute of Medical Science, University of Tokyo, Tokyo 108-8639, Japan;; ^b^Influenza Research Institute, Department of Pathobiological Sciences, School of Veterinary Medicine, University of Wisconsin−Madison, Madison, WI 53711;; ^c^Department of Molecular Virology, Research Institute for Microbial Diseases, Osaka University, Osaka 565-0871, Japan;; ^d^Department of Pathology, National Institute of Infectious Diseases, Tokyo 162-8640, Japan;; ^e^Department of Surgical Sciences, School of Veterinary Medicine, University of Wisconsin−Madison, Madison, WI 53706;; ^f^Division of Infectious Diseases, Advanced Clinical Research Center, Institute of Medical Science, University of Tokyo, Tokyo 108-8639, Japan;; ^g^Department of Infectious Diseases and Applied Immunology, IMSUT Hospital of The Institute of Medical Science, University of Tokyo, Tokyo 108-8639, Japan;; ^h^Emergency Medical Center, Fujisawa City Hospital, Kanagawa 251-8550, Japan;; ^i^Department of Pulmonary Medicine, Tokyo Saiseikai Central Hospital, Tokyo 108-0073, Japan;; ^j^Pulmonary Division, Department of Internal Medicine, Saiseikai Utsunomiya Hospital, Tochigi 321-0974, Japan;; ^k^Department of Emergency & Intensive Care, Saiseikai Utsunomiya Hospital, Tochigi 321-0974, Japan;; ^l^Department of Respiratory Medicine, Eiju General Hospital, Tokyo 110-8645, Japan;; ^m^Division of Infection Control, Eiju General Hospital, Tokyo 110-8645, Japan;; ^n^Infection Prevention and Control Department, Yokohama City University Hospital, Kanagawa 236-0004, Japan;; ^o^Department of Hematology and Clinical Immunology, Yokohama City University School of Medicine, Kanagawa 236-0004, Japan;; ^p^Department of Pulmonary Medicine, Keiyu Hospital, Kanagawa 220-8521, Japan;; ^q^Department of Refractory Viral Infections, National Center for Global Health and Medicine Research Institute, Tokyo 162-8655, Japan;; ^r^Disease Control and Prevention Center, National Center for Global Health and Medicine Hospital, Tokyo 162-8655, Japan;; ^s^Department of Pathology, University of Michigan, Ann Arbor, MI 48109;; ^t^Department of Pathology and Laboratory Medicine, University of Wisconsin−Madison, Madison, WI 53705;; ^u^Wisconsin National Primate Research Center, University of Wisconsin−Madison, Madison, WI 53715;; ^v^Department of Virology 1, National Institute of Infectious Diseases, Tokyo 208-0011, Japan;; ^w^Center for Emergency Preparedness and Response, National Institute of Infectious Diseases, Tokyo 162-8640, Japan;; ^x^Department of Veterinary Science, National Institute of Infectious Diseases, Tokyo 162-8640, Japan;; ^y^Department of Epidemiology, School of Public Health, University of Michigan, Ann Arbor, MI 48109;; ^z^Department of Pediatrics, Keiyu Hospital, Kanagawa 220-8521, Japan;; ^aa^Experimental Retrovirology Section, HIV and AIDS Malignancy Branch, National Cancer Institute, NIH, Bethesda, MD 20892;; ^bb^Department of Special Pathogens, International Research Center for Infectious Diseases, Institute of Medical Science, University of Tokyo, Tokyo 108-8639, Japan

**Keywords:** SARS-CoV-2, P.1 variant, Syrian hamsters, reinfection, convalescent human plasma

## Abstract

The spike (S) protein of severe acute respiratory syndrome coronavirus 2 (SARS-CoV-2) plays a key role in viral infectivity. It is also the major antigen stimulating the host's protective immune response, specifically, the production of neutralizing antibodies. Recently, a new variant of SARS-CoV-2 possessing multiple mutations in the S protein, designated P.1, emerged in Brazil. Here, we characterized a P.1 variant isolated in Japan by using Syrian hamsters, a well-established small animal model for the study of SARS-CoV-2 disease (COVID-19). In hamsters, the variant showed replicative abilities and pathogenicity similar to those of early and contemporary strains (i.e., SARS-CoV-2 bearing aspartic acid [D] or glycine [G] at position 614 of the S protein). Sera and/or plasma from convalescent patients and BNT162b2 messenger RNA vaccinees showed comparable neutralization titers across the P.1 variant, S-614D, and S-614G strains. In contrast, the S-614D and S-614G strains were less well recognized than the P.1 variant by serum from a P.1-infected patient. Prior infection with S-614D or S-614G strains efficiently prevented the replication of the P.1 variant in the lower respiratory tract of hamsters upon reinfection. In addition, passive transfer of neutralizing antibodies to hamsters infected with the P.1 variant or the S-614G strain led to reduced virus replication in the lower respiratory tract. However, the effect was less pronounced against the P.1 variant than the S-614G strain. These findings suggest that the P.1 variant may be somewhat antigenically different from the early and contemporary strains of SARS-CoV-2.

## Significance

Severe acute respiratory syndrome coronavirus 2 (SARS-CoV-2) variants are of concern, with the P.1 variants dominating in Brazil. Brazil is now seeing a record number of deaths. Here, we report that the pathogenicity in hamsters of a P.1 variant is similar to that of nonvariant SARS-CoV-2. However, it has an expanded host range as shown by its replication in mice. Prior infection with nonvariant SARS-CoV-2 strains efficiently prevented replication of the P.1 variant in the lower respiratory tract of hamsters upon reinfection. Convalescent sera from patients infected with nonvariants or sera from messenger RNA vaccinees showed comparable neutralization titers among the P.1 and previously circulating strains. These results suggest that previous SARS-CoV-2 infection and vaccines based on the original SARS-CoV-2 will provide some protection against P.1 infection.

Severe acute respiratory syndrome coronavirus 2 (SARS-CoV-2), which emerged as a novel human pathogen in China at the end of 2019, is responsible for COVID-19, which causes symptoms such as cough and fever, severe pneumonia, and death. The World Health Organization reported that, as of April 2021, ∼130 million cases of COVID-19 and 2.8 million associated deaths have occurred.

On January 6, 2021, Japan reported the detection of a new SARS-CoV-2 variant in travelers who arrived at Tokyo airport from Amazonas state, north Brazil (1, 2). This variant, designated P.1, is thought to have emerged in Brazil in November 2020 (3). As of April 2021, the P.1 variant has been detected in 36 countries, with local transmission occurring in 5 countries, including Brazil (4). The P.1 variant differs from early SARS-CoV-2 strains identified in Wuhan, China, by 12 amino acids in the spike (S) protein. S protein plays a key role in viral binding to host cell receptors (i.e., human angiotensin-converting enzyme 2 [hACE2]), and the P.1 variant has three mutations (K417T, E484K, and N501Y) in the receptor-binding domain (RBD). Previous studies suggest that both the E484K and N501Y mutations in the RBD may enhance the binding affinity of the S protein for hACE2 (5–7). In addition, the E484K substitution has been shown to confer resistance to monoclonal and polyclonal neutralizing antibodies in COVID-19 convalescent and postvaccination sera (8–12). However, the replicative capacity, pathogenicity, and antigenicity of the P.1 variant remain largely unknown. To better assess the risk posed by this variant, here we characterized isolates of the P.1 variant of SARS-CoV-2 in Japan in vitro and in vivo.

## Results and Discussion

To characterize the biological properties of the P.1 variant, we compared hCoV-19/Japan/TY7-501/2021 (TY7-501) (which was isolated from a traveler who arrived in Japan from Brazil) with SARS-CoV-2/UT-NCGM02/Human/2020/Tokyo (NCGM02) (13), an early SARS-CoV-2 strain from February 2020, and with SARS-CoV-2/UT-HP095-1N/Human/2020/Tokyo (HP095), which is genetically similar to contemporaneous SARS-CoV-2 strains that predominate globally. NCGM02 encodes aspartic acid (D) at amino acid position 614 of the S protein, whereas HP095 possesses a nonsynonymous mutation that encodes a D614G variant at this position. TY7-501 and HP-095 were propagated in the VeroE6 cell line VeroE6/TMPRSS2 (14), which constitutively expresses transmembrane protease serine 2 (TMPRSS2), which activates SARS-CoV-2 virus infection. NCGM02 was propagated in VeroE6 cells. Deep sequencing analysis of these virus stocks revealed that TY7-501 contained one addition mutation (G181V) at amino acid position 181 of the S protein (*SI Appendix*, Table S1). The G181V substitution, which is located in the N-terminal domain of the S protein, probably does not have a pivotal role in antigenic change, since amino acid substitutions at position 181 have never conferred resistance to neutralizing human monoclonal antibodies. In VeroE6/TMPRSS2 cells, TY7-501, NCGM02, and HP095 grew to similar titers (*SI Appendix*, Fig. S1).

**Fig. 1.**
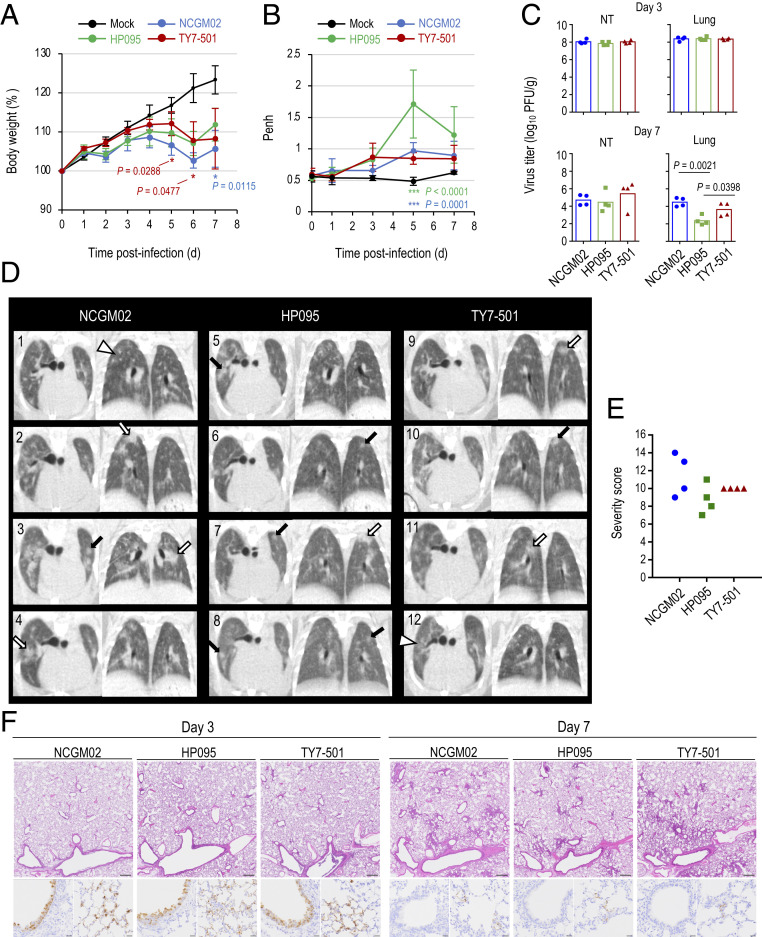
Virus replication and pathological findings in Syrian hamsters infected of the SARS-CoV-2 P.1 variant. Four Syrian hamsters per group were intranasally inoculated with 10^3^ PFU (in 30 μL) of hCoV-19/Japan/TY7-501/2021(TY7-501), SARS-CoV-2/UT-NCGM02/Human/2020/Tokyo (NCGM02), SARS-CoV-2/UT-HP095-1N/Human/2020/Tokyo (HP095), or phosphate-buffered saline (PBS) (mock). (*A*) Body weight changes in hamsters were monitored daily for 7 d after viral infection. Data are presented as the mean percentages of the starting weight (± SD). *P* values were calculated by using pairwise comparisons after a linear mixed model analysis (*P < 0.05). Red and blue asterisks indicate statistically significant differences between TY7-501−infected and NCGM02-infected animals and between NCGM02- and HP095-infected animals. (*B*) Pulmonary function analysis in infected hamsters. Penh, which is a surrogate marker for bronchoconstriction or airway obstruction, was measured by using WBP. Data are presented as the mean ± SD. *P* values were calculated by using pairwise comparisons after a linear mixed model analysis (***P < 0.001). Green and blue asterisks indicate statistically significant differences between TY7-501−infected and HP095-infected animals and NCGM02- and HP095-infected animals. (*C*) Syrian hamsters were euthanized on days 3 and 7 postinfection for virus titration. Virus titers in the nasal turbinates and lungs were determined by use of a plaque assay on VeroE6/TMPRSS2 cells. Vertical bars show the mean. Points indicate data from individual Syrian hamsters. NT, nasal turbinate. *P* values were calculated by using a one-way ANOVA, followed by Tukey’s post hoc test. (*D*) Micro-CT imaging of the lungs of infected hamsters on day 7 postinfection. Axial and dorsal/coronal plane CT images of the thorax in animals infected with NCGM02 (*1* to *4*), HP095 (*5* to *8*), or TY7-501 (*9* to *12*). Lung abnormalities included multifocal nodules (black arrows), ground glass opacity (white arrowheads), and regions of lung consolidation (white arrows) that were peripheral, bilateral, and multilobar. (*E*) CT severity score of TY7-501−infected, NCGM02-infected, and HP095-infected Syrian hamsters. NCGM02-infected animals had the highest CT severity score; HP095-infected animals had the lowest CT severity score. (*F*) Representative histopathological images of the lungs of hamsters infected with the indicated viruses on days 3 and 7 postinfection. (*Upper*) Hematoxylin/eosin (H&E) staining. (*Lower*) Immunohistochemistry (IHC) for SARS-CoV-2 antigen detection in bronchi (*Left*) and alveoli (*Right*). (Scale bars: H&E staining, 500 μm; IHC, 20 μm.)

We evaluated the replication and pathogenicity of the P.1 variant in Syrian hamsters, which are highly susceptible to SARS-CoV-2 (13, 15–17). Syrian hamsters were intranasally infected with 10^3^ plaque-forming units (PFU) of TY7-501, NCGM02, or HP095. The body weights of all of the Syrian hamsters infected with the three viruses gradually increased by day 4 or 5 postinfection (Fig. 1*A*). NCGM02-infected animals gained less weight than TY7-501−infected or HP095-infected animals. We also assessed pulmonary function in the infected hamsters by measuring enhanced pause (Penh), which is a surrogate marker for bronchoconstriction or airway obstruction, by using a whole-body plethysmography (WBP) system. All three viruses caused an increase in the lung Penh value on days 3, 5, and 7 postinfection (Fig. 1*B*). On day 5 postinfection, HP095-infected animals had significantly higher Penh values compared with those of TY7-501−infected or NCGM02-infected animals, but no differences were found between them on days 1, 3, and 7 postinfection. On day 3 postinfection, TY7-501, NCGM02, and HP095 replicated efficiently in the lungs and nasal turbinates of the infected animals, and no significant difference in viral replication was observed between the three groups (Fig. 1*C*). On day 7 postinfection, the lung titers in the HP095-infected group were significantly lower than those in the other two groups; no other marked differences in viral titers in the respiratory organs were found between the three groups.

Microcomputed tomography (micro-CT) analysis revealed lung abnormalities in all infected animals on day 7 postinfection that were consistent with commonly reported imaging features of COVID-19 pneumonia (18) (Fig. 1*D*). These lung abnormalities included multifocal nodular ground glass opacity with a peripheral, bilateral, multilobar, peribronchial distribution, and regions of lung consolidation. CT severity scores across all groups ranged from 7 to 14, with an overall average CT severity score of 10.1 (median 10) (Fig. 1*E*). NCGM02-infected animals had the highest CT severity score (mean 11.5 [range 9 to 14, median 11.5]), followed by TY7-501-infected animals (mean 10 [range 10 to 10, median 10]). HP095-infected animals had the lowest CT severity score (mean 8.75 [range 7 to 11, median 8.5]). Two of 12 animals developed a small-volume pneumomediastinum (one NCGM02-infected, and one HP095-infected), likely secondary to severe pulmonary damage, micropulmonary rupture, and gas tracking into the mediastinum.

Histopathological and immunohistochemical analyses were performed on the lung tissues of Syrian hamsters infected with NCGM02, HP095, or TY7-501 (Fig. 1*F*). Histopathology of the lung sections of the animals infected with NCGM02, HP095, or TY7-501 showed mild inflammatory cell infiltration around some bronchi without inflammation in the alveolar region on day 3 postinfection, and, by day 7 postinfection, focally extended inflammation from the peribronchial region to the alveoli was evident. There were no obvious differences in these histopathological findings among the virus strains. Immunohistochemistry revealed that viral antigens were present in the bronchial and alveolar epithelial cells of all of the virus-infected animals on day 3 postinfection; by day 7 postinfection, the number of viral antigen-positive cells had decreased, with only a few positive cells remaining in the alveolar region for all three virus strains.

Overall, these findings suggest that the replicative capacity and pathogenicity in hamsters of this P.1 variant of SARS-CoV-2 are similar to those of SARS-CoV-2 bearing D or glycine (G) at position 614 of the S protein.

**Fig. 2.**
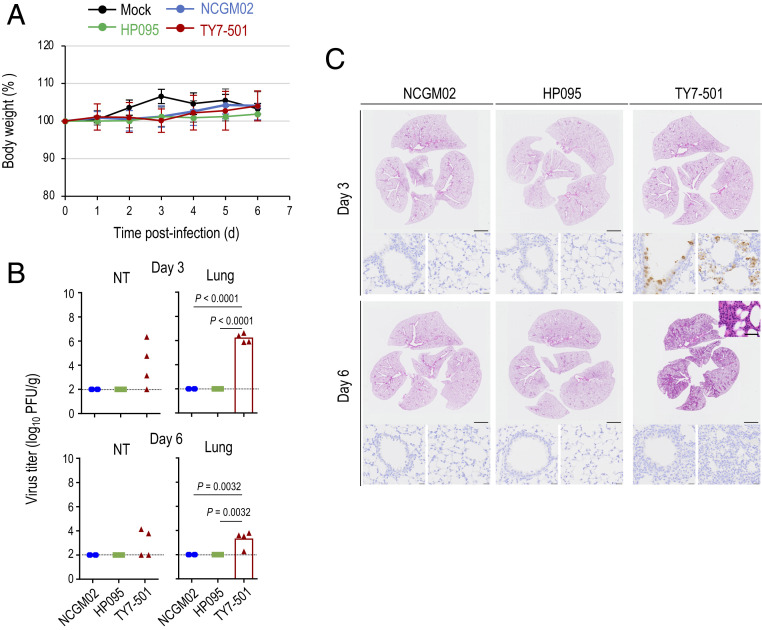
Virus replication and pathological findings in mice infected with the SARS-CoV-2 P.1 variant. Mice were intranasally inoculated with 10^5.7^ PFU (in 50 μL) of TY7-501, NCGM02, HP095, or PBS (mock). (*A*) Body weights of virus-infected (*n* = 7) and mock-infected mice (*n* = 7) were monitored daily for 6 d after viral infection. Data are presented as the mean percentages of the starting weight (±SD). *P* values were calculated by using pairwise comparisons after a linear mixed model analysis. (*B*) Four mice per group were euthanized on days 3 and 6 postinfection for virus titration. Virus titers were determined as described in the legend to Fig. 1. Vertical bars show the mean. The vertical bar is shown only when virus was recovered from all four mice. Points indicate data from individual mice. *P* values were calculated by using a one-way ANOVA, followed by Tukey’s post hoc test. The lower limit of detection is indicated by the horizontal dashed line. (*C*) Representative histopathological images of the lungs of mice infected with the indicated viruses on days 3 and 6 postinfection. (*Upper*) H&E staining. (Lower) IHC for SARS-CoV-2 antigen detection in bronchi (*Left*) and alveoli (*Right*). (*Inset*) higher magnification showing hyaline membrane formation in alveoli. (Scale bars: H&E staining, 2 mm; IHC, 20 μm; *Inset*, 20 μm.)

Mice are a useful small animal model for the evaluation of vaccines, immunotherapies, and antiviral drugs. Because the initial SARS-CoV-2 strains do not utilize murine ACE2 as a receptor (19), wild-type mice are not susceptible to SARS-CoV-2 infection (5, 20). However, studies have shown that the N501Y mutation in the RBD renders mice susceptible to SARS-CoV-2 infection (5, 21). This implies that P.1 variants with this mutation could replicate in the animal model. However, their replication capacity and pathogenicity in mice have not yet been determined. Therefore, we asked whether TY7-501 can replicate efficiently in the respiratory tract of mice. Intranasal inoculation of C57BL/6 mice with 10^5.7^ PFU of TY7-501, NCGM02, or HP095 did not cause changes in body weight in any group (Fig. 2*A*). However, we observed that TY7-501 replicated to high titers in the nasal turbinates and lungs of mice on day 3 postinoculation, although no virus was recovered from the nasal turbinate of one of the four animals on day 3 postinoculation, and no virus was recovered from the nasal turbinates of two of the four animals on day 6 postinoculation (Fig. 2*B*). In contrast, no virus was isolated from the respiratory organs of all four NCGM02- or HP095-infected animals on days 3 and 6 postinoculation. Histopathological and immunohistochemical analyses revealed that mice inoculated with NCGM02 or HP095 showed little inflammation, and no viral antigen was detected on either day 3 or day 6 postinoculation (Fig. 2*C*). By contrast, in mice inoculated with TY7-501, inflammatory cell infiltration was observed in the bronchi and peribronchial region on day 3 postinoculation, and extended to the alveolar region by day 6 postinoculation. Hyaline membrane formation was also observed in some alveoli (Fig. 2*C*, *Inset*). In addition, virus antigen was detected in the bronchial and alveolar epithelial cells on day 3 postinoculation, but not on day 6 postinoculation. Taken together, these observations suggest that mutations in the RBD of TY7-501, such as E484K and N501Y, may alter the binding affinity or specificity of the S protein for mouse cell surface molecules (including ACE2), thereby allowing the variant to replicate efficiently in mice.

**Fig. 3.**
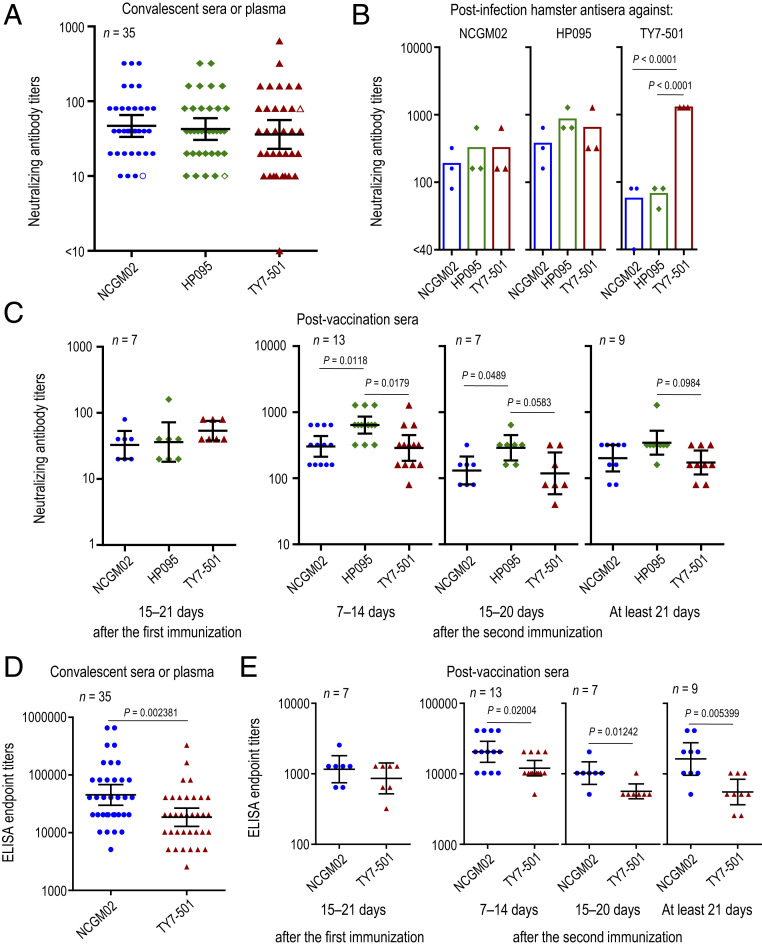
Antibody responses to the SARS-CoV-2 P.1 variant. (*A*) Neutralizing antibody titers of human sera or plasma obtained from recovered COVID-19 patients. Open symbols indicate the titers of serum from patient NCCo-503, who was infected with a P.1 variant. (*B*) Neutralizing antibody titers of postinfection hamster sera against SARS-CoV-2 variants. Syrian hamsters were intranasally inoculated with 10^3^ PFU of TY7-501, NCGM02, or HP095. Sera were collected on day 21 postinfection. (*C*) Neutralizing antibody titers in sera from BNT162b2 vaccinees. *P* values were calculated by using a one-way ANOVA, followed by Tukey’s post hoc test. (*D* and *E*) ELISA titers of antibodies against the RBD from TY7-501 or NCGM02 (*D*) in convalescent sera or plasma and (*E*) in sera from BNT162b2 vaccinees. *P* values were calculated by using a two-tailed unpaired Wilcoxon’s rank sum test with a continuity correction. Each dot represents data from one subject. Geometric mean titer and 95% CIs are shown.

Previous studies have shown that the E484K substitution in the RBD of the SARS-CoV-2 S protein confers resistance to monoclonal and polyclonal neutralizing antibodies in COVID-19 convalescent and postvaccination sera (8–12), suggesting that P.1 variants with this mutation may be antigenically different from contemporary SARS-CoV-2. To assess the antigenic characteristic of P.1 variants, we examined the neutralizing ability of convalescent human sera or plasma obtained from 35 COVID-19 patients against TY7-501, NCGM02, and HP095. All of the convalescent human sera and plasma showed comparable neutralization titers among the viruses tested, except for serum from patient NCCo-503, who was the only individual infected with the P.1 variant (Fig. 3*A* and Table 1). A similar trend was observed for sera collected from hamsters infected with these viruses (Fig. 3*B*). Antisera raised against NCGM02 or HP095 exhibited similar neutralization titers across the three strains. However, NCGM02 and HP095 were poorly recognized by the hamster antisera raised against TY7-501, with titers 16- or 32-fold lower than the homologous titer. These results suggest that the P.1 variant may be antigenically different from the early and contemporary strains of SARS-CoV-2.

**Table 1.** Neutralizing antibody titers of convalescent human sera or plasma against SARS-CoV-2 variants

Patient ID	Age, y	Onset day	Collection day	Days from onset	Sample type	Neutralizing antibody titer		
						NCGM02	HP095	TY7-501
HPCo-010	55	3/1/2020	6/3/2020	94	Plasma	10	10	20
HPCo-015	49	3/16/2020	6/18/2020	94	Plasma	10	10	10
HPCo-022	67	3/18/2020	6/25/2020	99	Plasma	20	20	10
HPCo-025	52	4/1/2020	7/3/2020	93	Plasma	20	20	10
HPCo-029	61	3/29/2020	6/29/2020	92	Plasma	80	40	40
FSCo-001	75	2/8/2020	2/29/2020	21	Serum	40	40	80
FSCo-002	67	2/3/2020	7/6/2020	154	Serum	20	20	20
FSCo-003	80	2/14/2020	3/19/2020	34	Serum	40	40	10
FSCo-004	64	3/29/2020	5/3/2020	35	Serum	320	160	160
FSCo-005	62	4/1/2020	5/18/2020	47	Serum	40	20	10
FSCo-006	77	5/23/2020	7/3/2020	41	Serum	40	80	40
STCo-001	47	3/25/2020	4/10/2020	16	Serum	20	10	<10
STCo-002	38	3/23/2020	4/14/2020	22	Serum	160	160	160
STCo-003	68	3/28/2020	4/20/2020	23	Serum	160	80	80
STCo-005	59	4/3/2020	7/9/2020	97	Plasma	20	10	10
STCo-006	38	4/24/2020	5/25/2020	31	Plasma	40	40	20
STCo-007	64	4/4/2020	4/28/2020	24	Serum	40	40	20
STCo-008	46	3/29/2020	6/3/2020	66	Plasma	40	40	40
STCo-009	79	4/11/2020	7/7/2020	87	Plasma	80	40	40
SUCo-001	57	3/5/2020	3/31/2020	33	Plasma	80	160	160
SUCo-001	57	3/5/2020	5/20/2020	76	Plasma	80	40	20
SUCo-002	60	3/21/2020	4/23/2020	26	Plasma	320	320	640
SUCo-002	60	3/21/2020	5/7/2020	47	Serum	80	80	40
SUCo-004	81	4/16/2020	6/1/2020	46	Serum	320	320	320
SUCo-005	73	4/1/2020	5/28/2020	57	Serum	40	40	10
EJCo-001	69	1/28/2020	2/18/2020	21	Serum	160	160	80
EJCo-002	60	2/12/2020	2/25/2020	13	Serum	20	20	20
EJCo-003	78	2/10/2020	2/25/2020	15	Serum	40	40	80
EJCo-004	70	2/20/2020	3/2/2020	11	Serum	10	20	10
HICo-002	64	2/3/2020	6/5/2020	123	Plasma	80	80	160
HICo-002	64	2/3/2020	7/3/2020	151	Plasma	40	40	40
HICo-004	67	2/24/2020	5/29/2020	95	Plasma	80	80	160
KYCo-001	50	4/4/2020	7/9/2020	96	Plasma	20	20	20
KYCo-002	65	4/9/2020	6/4/2020	56	Plasma	80	80	80
NCCo-503	48	1/2/2021	1/22/2021	20	Serum	10	10	80

Dates are given as month/day/year.

We next examined the reactivity of sera from individuals who received the messenger RNA (mRNA) vaccine BNT162b2 (Pfizer-BioNTech) against TY7-501, NCGM02, and HP095 (Fig. 3*C* and Table 2). Most of the sera from individuals immunized with this vaccine showed similar neutralization titers between TY7-501 and NCGM02. This finding is consistent with a previous report that the amino acid mutations found in the S protein of P.1 variants did not affect the neutralizing ability of human sera elicited by this mRNA vaccine when tested against live SARS-CoV-2 strains (22). Our data also showed that the neutralization titer of the serum panel at 7 d to 14 d and/or 15 d to 20 d after the second immunization against HP095 was slightly higher than the neutralization titers against TY7-501 and NCGM02. We also determined titers of RBD-specific IgG antibodies in the blood samples from convalescent individuals and BNT162b2 vaccinees, using ELISAs coated with the recombinant RBD derived from NCGM02 or TY7-501. Compared to the RBD derived from NCGM02, most of the convalescent sample showed twofold to fourfold reduced titer against the TY7-501 RBD (Fig. 3*D* and *SI Appendix*, Table S2). Similar results were found in the serum panel after the immunization (Fig. 3*E* and *SI Appendix*, Table S3). Together, these results may suggest that the current COVID-19 mRNA vaccines confer somewhat similar levels of protection against P.1 variants as for other SARS-CoV-2 strains.

**Table 2.** Neutralizing antibody titers of sera from BNT162b2 vaccinees against SARS-CoV-2 variants

Vaccinated human sera	Sample ID	Neutralizing antibody titer		
		NCGM02	HP095	TY7-501
15 d to 21 d after the first immunization	1785	40	20	80
	2946	20	20	40
	9163	40	40	40
	2955	20	40	80
	8787	20	20	40
	8832	40	40	40
	11182	80	160	80
7 d to 14 d after the second immunization	7482	320	640	320
	11462	640	1,280	1,280
	1610	160	640	80
	4500	160	640	320
	1497	320	640	320
	4719	160	320	160
	4704	160	640	640
	7635	160	640	320
	7533	640	1,280	640
	4608	640	640	160
	7629	320	320	160
	7854	640	1,280	320
	11471	320	320	160
15 d to 20 d after the second immunization	7806	320	640	320
	7644	160	320	320
	7455	80	320	80
	4605	160	320	80
	7683	80	160	80
	7602	160	160	160
	9418	80	320	40
At least 21 d after the second immunization	7656	160	320	320
	7614	160	320	160
	7794	320	1,280	320
	9394	320	320	160
	7626	320	320	160
	7686	80	320	80
	7641	80	160	80
	7701	320	320	160
	7836	320	320	320

**Fig. 4.**
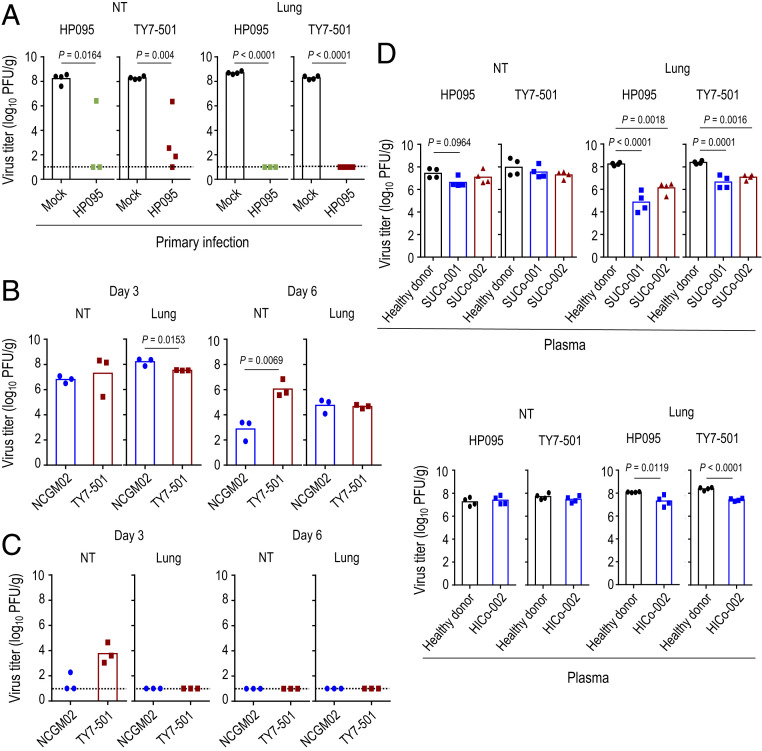
Inhibitory effects of neutralizing antibodies on the replication of the SARS-CoV-2 P.1 variant. (*A*) Syrian hamsters were intranasally reinfected with 10^3^ PFU (in 30 μL) of TY7-501 or HP095 at 21 d after primary infection with HP095. Four or three Syrian hamsters per group were euthanized on day 4 after rechallenge for virus titration. The lower limit of detection is indicated by the horizontal dashed line. (*B*) Virus replication in infected hamsters. Syrian hamsters were intranasally inoculated with 10^3^ PFU (in 30 μL) of NCGM02 or TY7-501. Three hamsters per group were euthanized on days 3 and 6 postinfection for virus titration. (*C*) Syrian hamsters were intranasally reinfected with 10^3^ PFU (in 30 μL) of TY7-501 or NCGM02 9 mo after primary infection with NCGM02. Three Syrian hamsters per group were euthanized on days 3 and 6 after rechallenge for virus titration. The lower limit of detection is indicated by the horizontal dashed line. (*D*) Effect of convalescent plasma on the replication of the P.1 variant. Syrian hamsters were inoculated intranasally with 10^3^ PFU of TY7-501 or HP095. On day 1 postinfection, the hamsters were injected intraperitoneally with plasma from COVID-19 patients or pooled plasma from healthy donors. Animals were euthanized on day 4 postinfection for virus titration. Virus titers were determined as described in the legend to Fig. 1. Vertical bars show the mean. The vertical bar is shown only when virus was recovered from all (*A* and *D*) four or (*B* and *C*) three hamsters. Points indicate data from individual hamsters. *P* values were calculated by using a one-way ANOVA, followed by Tukey’s post hoc test.

Are individuals who were previously infected with SARS-CoV-2 bearing S-614D or S-614G protected against P.1 variants? To address this question, we examined whether hamsters that develop antibodies against HP095 are resistant to subsequent reinfection with TY7-501. Four or three hamsters that were previously infected with 10^3^ PFU of HP095 were rechallenged with 10^3^ PFU of HP095 or TY7-501 21 d after the primary infection. On day 4 postreinfection, in the mock-infected control group, high virus titers were detected in the respiratory tract of all four HP095-infected or TY7-501−infected animals (Fig. 4*A*). By contrast, in the group previously infected with HP095, no virus was detected in the lungs of all seven animals that were reinfected with HP095 or TY7-501. However, viruses were recovered from the nasal turbinates of three of four animals reinfected with TY7-501 and from the nasal turbinates of one of three animals reinfected with HP095. Neutralization assays with sera collected 20 d after the primary infection revealed that all of the infected animals had seroconverted (*SI Appendix*, Table S4).

To evaluate the long-term durability of immunity elicited by prior infection and its protective effect against a reinfection with the P.1 variant, hamsters were intranasally reinfected with 10^3^ PFU of TY7-501 or NCGM02 9 mo after the primary infection with NCGM02. First, we compared the replicative ability of TY7-501 with that of NCGM02 in age-matched Syrian hamsters (Fig. 4*B*). The two viruses replicated in the lungs of the infected animals with no difference in mean virus titers on day 6 postinfection; however, the mean virus titer of NCGM02 in the lungs (mean titer = 8.2 log_10_ [PFU/g]) was significantly higher than that of TY7-501 (mean titer = 7.5 log_10_ [PFU/g]) on day 3 postinfection. For reinfection experiments, we used hamsters previously infected with 1, 10, 10^2^, or 10^3^ PFU of NCGM02, and measured their ELISA and neutralizing antibody titers against the NCGM02 S protein and NCGM02 virus, respectively, in sera collected 9 mo after the primary infection (*SI Appendix*, Table S5). We divided the hamsters into two groups based on their antibody titers so that each group contained animals with similar antibody titers. Even 9 mo after the initial infection, previous infection with NCGM02 provided a high level of resistance to the replication of these two viruses in the lungs upon reinfection (Fig. 4*C*). However, in the nasal turbinates, viruses were recovered from one of three animals reinfected with the homologous virus and from all three animals reinfected with TY7-501. Taken together, these observations suggest that neutralizing antibodies elicited by prior infection efficiently restrict viral replication during subsequent infection with P.1 variants in the lower respiratory tract but not in the upper respiratory organs. However, it should be noted that the amounts of P.1 virus in the upper respiratory organs were ∼1,000-fold lower in reinfected animals compared with those in naïve animals, indicating that, even in the upper respiratory organs, animals previously infected with NCGM02 are somewhat protected against reinfection with the P.1 virus.

We next assessed the protective efficacy of convalescent plasma from COVID-19 patients on the replication of the P.1 variant in the respiratory tract of hamsters. Postinfection plasma was collected from three COVID-19 patients (SUCo-001, SUCo-002, and HICo-002) who had developed respiratory symptoms on March 5, March 21, and February 3, 2020, respectively (Table 1). Plasma from each of the patients was then transferred intraperitoneally to four hamsters on day 1 after infection with 10^3^ PFU of HP095 or TY7-501. Pooled normal plasma obtained from 10 healthy donors was injected intraperitoneally into four hamsters on day 1 postinfection as a control. For both the HP095-infected and TY7-501−infected groups, no differences in viral titers in the nasal turbinates were observed between the animals that received postinfection plasma and the animals that received normal plasma (Fig. 4*D*). In contrast, virus titers in the lungs of the animals that received postinfection plasma were significantly lower than those in the organs of the animals that received normal plasma. No substantial difference in the levels of virus titer reduction in the lungs of the animals that received plasma from the patient HICo-002 was observed between HP095-infected and TY7-501−infected groups (mean reduction in viral titer = 0.8 and 1.0 log_10_ [PFU/g], respectively). However, in the lungs of the animals that received plasma from the other two patients (SUCo-001 and SUCo-002), the levels of virus titer reduction were greater in the HP095-infected group (3.4 and 2.1 log_10_ [PFU/g] for SUCo-001 and SUCo-002, respectively) than in the TY7-501−infected group (1.7 and 1.3 log_10_ [PFU/g] for SUCo-001 and SUCo-002, respectively), although these differences were not statistically significant.

The emergence of SARS-CoV-2 P.1 variants carrying mutations in the RBD of the S protein has raised concern that these variants could escape the immunity elicited by previous infection or vaccination. In this study, we observed that early and contemporary strains (i.e., SARS-CoV-2 bearing D or G at position 614 of the S protein) and the P.1 variant were similarly neutralized by sera and/or plasma from convalescent COVID-19 patients and individuals who received a currently authorized vaccine. In contrast, the S-614D or S-614G strains were less well recognized than the P.1 variant by the serum from a P.1-infected patient. Prior infection with S-614D or S-614G strains efficiently prevented the replication of the P.1 variant in the lower respiratory tract of hamsters upon reinfection. In addition, passive transfer of neutralizing antibodies to hamsters infected with the P.1 variant or the S-614G strain led to reduced virus replication in the lower respiratory tract. However, the effect was less pronounced against the P.1 variant than the S-614G strain. These findings suggest that the P.1 variant may be somewhat antigenically different from the early and contemporary strains of SARS-CoV-2. Therefore, the spread of this P.1 variant should be monitored very closely.

## Materials and Methods

### Clinical Specimens.

After informed consent was obtained, specimens were collected from individuals with and without SARS-CoV-2 infection. The research protocol was approved by the Research Ethics Review Committee of the Institute of Medical Science of the University of Tokyo (approval number 2019–71–0201).

After informed consent was obtained, specimens were collected from individuals who received the mRNA vaccine BNT162b2 (Pfizer-BioNTech) in the Immunity Associated with SARS-CoV-2 (IASO) cohort study. The research protocol was approved by the institutional review board at the University of Michigan Medical School (protocol number HUM00184533).

### Viruses.

HP095 and TY7-501 were propagated in VeroE6/TMPRSS2 cells in VP-SFM (Thermo Fisher Scientific). NCGM02 has been previously described (13). All experiments with SARS-CoV-2 viruses were performed in enhanced biosafety level 3 containment laboratories as described previously (13).

### Animal Experiments.

All animal experiments were performed in accordance with the Science Council of Japan’s Guidelines for Proper Conduct of Animal Experiments and the guidelines set by the Institutional Animal Care and Use Committee at the University of Wisconsin−Madison as described elsewhere (13).

Detailed materials and methods for this study are described in *SI Appendix*.

## Supplementary Material

Supplementary File

## Data Availability

All data supporting the findings of this study are included in the main text and *SI Appendix*; any materials will be made available upon reasonable request.
