# Supplementary material for: Characterization of a new SARS-CoV-2 variant that emerged in Brazil
Source: Proc Natl Acad Sci U S A. 2021 Jun 17;118(27):e2106535118. doi: 10.1073/pnas.2106535118 (PMC8271735; doi:10.1073/pnas.2106535118)
Supplement: Supplementary File [file pnas.2106535118.sapp.pdf]

PNAS
www.pnas.org

Supplementary Information for

Characterization of a new SARS-CoV-2 variant that emerged in Brazil

Masaki Imai[1,29], Peter J. Halfmann[2,29], Seiya Yamayoshi[1,29], Kiyoko Iwatsuki-Horimoto[1], Shiho Chiba[2], Tokiko Watanabe[1,3], Noriko Nakajima[4], Mutsumi Ito[1], Makoto Kuroda[2], Maki Kiso[1], Tadashi Maemura[1,2], Kenta Takahashi[4], Samantha Loeber[5], Masato Hatta[2], Michiko Koga[6,7], Hiroyuki Nagai[7], Shinya Yamamoto[6,7], Makoto Saito[6,7], Eisuke Adachi[7], Osamu Akasaka[8], Morio Nakamura[9], Ichiro Nakachi[10], Takayuki Ogura[11], Rie Baba[10], Kensuke Fujita[11], Junichi Ochi[12], Keiko Mitamura[13], Hideaki Kato[14,15], Hideaki Nakajima[15], Kazuma Yagi[16], Shin-ichiro Hattori[17], Kenji Maeda[17], Tetsuya Suzuki[18], Yusuke Miyazato[18], Riccardo Valdez[19], Carmen Gherasim[19], Yuri Furusawa[1], Moe Okuda[1], Michiko Ujie[1], Tiago J. S. Lopes[1,2], Atsuhiro Yasuhara[1], Hiroshi Ueki[1], Yuko Sakai-Tagawa[1], Amie J. Eisfeld[2], John J. Baczenas[20,21], David A. Baker[20], Shelby L. O'Connor[20,21], David H. O'Connor[20,21], Shuetsu Fukushi[22], Tsuguto Fujimoto[23], Yudai Kuroda[24], Aubree Gordon[25], Ken Maeda[24], Norio Ohmagari[18], Norio Sugaya[26], Hiroshi Yotsuyanagi[6,7], Hiroaki Mitsuya[17,27], Tadaki Suzuki[4], Yoshihiro Kawaoka[1,2,28]

[1]*Division of Virology, Department of Microbiology and Immunology, Institute of Medical Science, University of Tokyo, Tokyo 108-8639, Japan*
[2]*Influenza Research Institute, Department of Pathobiological Sciences, School of Veterinary Medicine, University of Wisconsin-Madison, WI 53711, USA*
[3]*Department of Molecular Virology, Research Institute for Microbial Diseases, Osaka University, Osaka 565-0871, Japan*
[4]*Department of Pathology, National Institute of Infectious Diseases, Tokyo 162-8640, Japan*
[5]*Department of Surgical Sciences, School of Veterinary Medicine, University of Wisconsin-Madison, WI 53706, USA*
[6]*Division of Infectious Diseases, Advanced Clinical Research Center, Institute of Medical Science, University of Tokyo, Tokyo 108-8639, Japan*
[7]*Department of Infectious Diseases and Applied Immunology, IMSUT Hospital of The Institute of Medical Science, the University of Tokyo, Tokyo 108-8639, Japan*
[8]*Emergency Medical Center, Fujisawa City Hospital, Kanagawa 251-8550, Japan*
[9]*Department of Pulmonary Medicine, Tokyo Saiseikai Central Hospital, Tokyo 108-0073, Japan*
[10]*Pulmonary division, Department of Internal Medicine, Saiseikai Utsunomiya Hospital, Tochigi 321-0974, Japan*
[11]*Department of Emergency & Intensive care, Saiseikai Utsunomiya Hospital, Tochigi 321-0974, Japan*
[12]*Department of Respiratory Medicine, Eiju General Hospital, Tokyo 110-8645, Japan*
[13]*Division of Infection Control, Eiju General Hospital, Tokyo 110-8645, Japan*
[14]*Infection Prevention and Control Department, Yokohama City University Hospital, Kanagawa 236-0004, Japan*
[15]*Department of Hematology and Clinical Immunology, Yokohama City University School of Medicine, Kanagawa 236-0004, Japan*
[16]*Department of Pulmonary Medicine, Department of Medicine, Keiyu Hospital, Kanagawa 220-8521, Japan*
[17]*Department of Refractory Viral Infections, National Center for Global Health and Medicine Research Institute, Tokyo 162-8655, Japan*

[18]*Disease Control and Prevention Center, National Center for Global Health and Medicine Hospital, Tokyo 162-8655, Japan*
[19]*Department of Pathology, University of Michigan, Ann Arbor, MI 48109, USA.*
[20]*Department of Pathology and Laboratory Medicine, University of Wisconsin-Madison, Madison, WI 53705, USA*
[21]*Wisconsin National Primate Research Center, University of Wisconsin-Madison, Madison, WI 53715, USA*
[22]*Department of Virology 1, National Institute of Infectious Diseases, Musashimurayama, Tokyo 208-0011, Japan.*
[23]*Center for Emergency Preparedness and Response, National Institute of Infectious Diseases, Tokyo 162-8640, Japan*
[24]*Department of Veterinary Science, National Institute of Infectious Diseases, Tokyo 162-8640, Japan*
[25]*Department of Epidemiology, School of Public Health, University of Michigan, Ann Arbor, MI 48109, USA*
[26]*Department of Pediatrics, Keiyu Hospital, Kanagawa 220-8521, Japan*
[27]*Experimental Retrovirology Section, HIV & AIDS Malignancy Branch, National Cancer Institute, National Institutes of Health, Bethesda, MD 20892, USA.*
[28]*Department of Special Pathogens, International Research Center for Infectious Diseases, Institute of Medical Science, University of Tokyo, Tokyo 108-8639, Japan*
[29]*These authors contributed equally*

Yoshihiro Kawaoka
Email: yoshihiro.kawaoka@wisc.edu

This PDF file includes:

Supplementary methods
Figure S1
Tables S1 to S5
SI References

Supplementary methods

Cells. VeroE6/TMPRSS2 (1) (JCRB 1819) cells were propagated in the presence of 1 mg/ml geneticin (G418; Invivogen) and 5 μg/ml plasmocin prophylactic (Invivogen) in DMEM containing 10% FCS and antibiotics. The cells were incubated at 37 °C with 5% CO_2, regularly tested for mycoplasma contamination by using PCR, and confirmed to be mycoplasma-free.

Clinical specimens. After informed consent was obtained, specimens were collected from individuals with and without SARS-CoV-2 infection. The research protocol was approved by the Research Ethics Review Committee of the Institute of Medical Science of the University of Tokyo (approval number 2019–71–0201).

After informed consent was obtained, specimens were collected from individuals who received the mRNA vaccine BNT162b2 (Pfizer-BioNTech) in the Immunity-associated with SARS-CoV-2 (IASO) cohort study. The research protocol was approved by the institutional review board at the University of Michigan Medical School (protocol number HUM00184533.

Viruses. HP095 and TY7-501 were propagated in VeroE6/TMPRSS2 cells in VP-SFM (Thermo Fisher Scientific). NCGM02 (2) was propagated in VeroE6 cells in Opti-MEM I (Thermo Fisher Scientific) containing 0.3% bovine serum albumin (BSA) and 1 μg of L-1-Tosylamide-2-phenylethyl chloromethyl ketone (TPCK)-trypsin/ml.

All experiments with SARS-CoV-2 viruses were performed in enhanced biosafety level 3 (BSL3) containment laboratories at the University of Tokyo, which are approved for such use by the Ministry of Agriculture, Forestry, and Fisheries, Japan, or in enhanced BSL3 containment laboratories at the University of Wisconsin-Madison, which are approved for such use by the Centers for Disease Control and Prevention and by the US Department of Agriculture.

Multiplex PCR for SARS-CoV-2 genomes. SARS-CoV-2 inocula were sequenced using a modified approach originally developed by ARTIC Network (https://artic.network/ncov-2019). Briefly, complementary DNA (cDNA) was synthesized using SuperScript IV Reverse Transcriptase (Invitrogen, Carlsbad, CA, USA), random hexamers, and dNTPs. The cDNA was then PCR-amplified following a multiplex PCR amplicon-based approach that was developed for Nanopore (3,4). Briefly, 96 primers were used to produce cDNA amplicons that span the entire length of the genome (3). Two multiplexed PCR reactions with Q5 Hot Start Hi-Fi 2x Master mix (New England Biolabs, Ipswich, MA, USA) and cDNA as the starting template. The following thermocycling conditions were used: 98°C for 30 seconds, followed by 25 cycles of 98°C for 15 seconds and. 65°C for five minutes, then followed by an indefinite hold at 4°C (3,4). Once amplification was complete, the samples were pooled together before proceeding into the Illumina TruSeq library prep.

TruSeq Illumina library prep and sequencing. The pooled, amplified cDNA was purified with AMPure XP beads (Beckman Coulter, Brea, CA, USA). Each sample was then processed and prepped for deep sequencing using the TruSeq sample preparation kit (Illumina, USA). The samples were end repaired and then purified using the Sample Purification Beads (SPB). Each sample was then A-Tailed by attaching a non-templated nucleotide to the 3' end, followed by an adaptor ligation phase. A post ligation bead cleanup, using SPB was performed. Finally, each sample was amplified via eight cycles of PCR, followed by a bead cleanup using SPB, and eluted in RSB. The concentration and average fragment length were determined with a Qubit dsDNA high-sensitivity kit (Invitrogen, USA) and Agilent's High Sensitivity DNA kit, respectively. Each sample was pooled equimolarly to a concentration of 4 nM. This pool was denatured with five μL of 0.2 N NaOH, vortexed, and incubated at room temperature for five minutes. HT1 buffer solution was added to generate a 20 pM pool. The 20pM pool was then diluted to a final concentration of 10 pM and a Phix-derived control was spiked in, to account for 10% of the total DNA. The pool was loaded onto a 2 X 250 cycle V2 cartridge, to be sequenced on an Illumina MiSeq.

Bioinformatic analysis of raw sequencing data. An analytical pipeline called "Zequencer V7" was using to process the raw FASTQ files. In short, the primer sequences were trimmed and the reads were paired and merged using BBDuk (https://jgi.doe.gov/data-and-tools/bbtools/bb-tools-user-guide/bbduk-guide/) and BBMerge (https://jgi.doe.gov/data-and-tools/bbtools/bb-tools-user-guide/bbmerge-guide/). The reads were then mapped to the reference (MN908947.3) using BBMap (https://jgi.doe.gov/data-and-tools/bbtools/bb-tools-user-guide/bbmap-guide/). Variants were called using var2.CallVariants (embedded in BBMap) and annotated via samtools 1.10 (https://github.com/samtools/samtools/releases/). The VCF files were cleaned and converted into variant tables using

a custom Python script, that can be found on Github (https://github.com/katarinabraun/SARSCoV2_transmission_in_domestic_cats/blob/main/code/data_cleaning_and_intersection_plots.ipynb). Variants were called at ≥10% in reads that were ≥100 bp in length.

Animal experiments. The sample sizes for the hamster and mouse studies were chosen because they have previously been shown to be sufficient to evaluate a significant difference among groups (5-7). No method of randomization was used to allocate the animals to the experimental groups in this study. The investigators were not blinded to the group allocation during the experiments or when assessing the outcome.

All experiments with hamsters and mice were performed in accordance with the Science Council of Japan's Guidelines for Proper Conduct of Animal Experiments and the guidelines set by the Institutional Animal Care and Use Committee at the University of Wisconsin-Madison. The protocols were approved by the Animal Experiment Committee of the Institute of Medical Science, the University of Tokyo (approval numbers PA19-72 and PA19-75) and the Animal Care and Use Committee of the University of Wisconsin-Madison (protocol number V6426).

Growth kinetics of virus in cell culture. Cell cultures of VeroE6/TMPRSS2 grown on 12-well plates were infected in triplicate with TY7-501, NCGM02, and HP095 at a multiplicity of infection (MOI) of 0.001. After infection, the cells were incubated at 37 °C in DMEM containing 5% FCS. Cell supernatants were collected at 3, 24, 48, and 72 h post-infection, and virus titers in the cell supernatants were determined by use of a plaque assay on VeroE6/TMPRSS2 cells.

Experimental infection of Syrian hamsters. One-month-old male Syrian hamsters (Japan SLC Inc., Shizuoka, Japan) and nine-month-old female Syrian hamsters (Envigo, Indianapolis, IN, USA) were used in this study. Baseline body weights were measured before infection. Under *isoflurane* anesthesia, three or four hamsters per group were intranasally inoculated with 10^3 PFU (in 30 μl) of NCGM02, HP095, or TY7-501. Body weight was monitored daily for 7 days. For virological and pathological examinations, three or four hamsters per group were intranasally infected with 10^3 PFU of the viruses; 3, 6, and/or 7 days post-infection, the animals were euthanized and nasal turbinates and lungs were collected. The virus titers in the nasal turbinates and lungs were determined by use of plaque assays on VeroE6/TMPRSS2 cells.

For the re-infection experiments after initial infection with HP095, eight hamsters per group were intranasally inoculated with 10^3 PFU (in 30 μl) of HP095 or PBS (mock). On Day 21 post-infection, four animals per group were re-infected with 10^3 PFU (in 30 μl) of HP095 or TY7-501. On Day 3 after re-infection, the animals were euthanized, and the virus titers in the nasal turbinates and lungs were determined by performing plaque assays on VeroE6/TMPRSS2 cells.

For the re-infection experiments after initial infection with NCGM02, six hamsters per group were intranasally inoculated with 10^3 PFU (in 30 μl) of NCGM02. At 9 months post-infection, three animals per group were re-infected with 10^3 PFU (in 30 μl) of NCGM02 or TY7-501; on Days 3 and 6 post-infection, the animals were euthanized and their nasal turbinates and lungs were collected. The virus titers in the nasal turbinates and lungs were determined by use of plaque assays on VeroE6/TMPRSS2 cells.

For the passive transfer experiments, four hamsters per group were inoculated intranasally with 10^3 PFU (in 30 μl) of HP095 or TY7-501. On Day 1 post-infection, the hamsters were injected intraperitoneally with post-infection serum or control serum (1 ml per hamster). The animals were euthanized on Day 4 post-infection, and the virus titers in the nasal turbinates and lungs were determined by use of plaque assays on VeroE6/TMPRSS2 cells.

Experimental infection of mice. Six-week-old female C57BL/6 mice (Japan SLC Inc., Shizuoka, Japan) were used in this study. Baseline body weights were measured before infection. Under isoflurane anesthesia, seven mice per group were intranasally inoculated with $10^{5.7}$ PFU (in 50 μl) of NCGM02, HP095, or TY7-501. Body weight was monitored daily for 6 days. For virological and pathological examinations, eight mice per group were intranasally inoculated with $10^{5.7}$ PFU (in 50 μl) of the viruses and four mice per group were euthanized at 3 and 6 days post-infection. The virus titers in various organs were determined by use of plaque assays on VeroE6/TMPRSS2 cells.

Lung function. Respiratory parameters were measured by using a whole-body plethysmography system (PrimeBioscience) according to the manufacturer's instructions. In brief, hamsters were placed in the unrestrained plethysmography chambers and allowed to acclimatize for 1 min before data were acquired over a 3-min period by using FinePointe software.

Micro-CT imaging. One-month-old male Syrian hamsters (Japan SLC Inc., Shizuoka, Japan) were used in this study. Four hamsters per group were intranasally inoculated with 10^3 PFU (in 30 µl) of NCGM02, HP095, or TY7-501, respectively. Respiratory organs of the infected animals were imaged by using an *in vivo* micro-CT scanner (CosmoScan FX; Rigaku Corporation, Japan) on Day 7 post-infection. Under ketamine-xylazine anesthesia, the animals were placed in the image chamber and were scanned for 2 min at 90 kV, 88 µA, FOV 45 mm, and pixel size 90.0 µm. After scanning, the lung images were reconstructed by using the CosmoScan Database software of the micro-CT (Rigaku Corporation, Japan) and analyzed by using the manufacturer-supplied software.

Qualitative and semiquantitative visual image analysis of the lungs was performed in 12 male Syrian Hamsters at pre-infection and Day 7 post-infection with NCGM02 (n=4), HP095 (n=4), or TY7-501 (n=4). A CT severity score, adapted from a human scoring system, was used to grade the severity of the lung abnormalities (8). Each lung lobe was analyzed for degree of involvement and scored from 0–4 depending on the severity: 0 (none, 0%), 1 (minimal, 1%–25%), 2 (mild, 26%–50%), 3 (moderate, 51%–75%), or 4 (severe, 76%–100%). Scores for the five lung lobes were summed to obtain a total severity score of 0–20, reflecting the severity of abnormalities across the three infected groups. Images were anonymized and randomized; the scorer was blinded to the group allocation.

Pathological examination. Excised animal tissues were fixed in 4% paraformaldehyde phosphate buffer solution, and processed for paraffin embedding. The paraffin blocks were cut into 3-µm-thick sections and then mounted on silane-coated glass slides. One section from each tissue sample was stained using a standard hematoxylin and eosin procedure; another was processed for immunohistochemical staining with a rabbit polyclonal antibody for SARS-CoV-1 virus nucleocapsid protein (Prospec; ANT-180), which cross-reacts with SARS-CoV-2 nucleocapsid protein. Specific antigen-antibody reactions were visualized by means of 3,3'-diaminobenzidine tetrahydrochloride staining using the Dako Envision system (Dako Cytomation).

Virus neutralization assay. Human blood samples (serum or plasma) were incubated at 56 °C for at least 30 min. Thirty-five microliters of virus (140 tissue culture infectious dose 50) was incubated with 35 µl of two-fold serial dilutions of serum or plasma for 1 h at room temperature, and 50 µl of the mixture was added to confluent VeroE6/TMPRSS2 cells in 96-well plates, and incubated for 1 h at 37 °C. After the addition of 50 µl of DMEM containing 5% FCS, the cells were incubated for 3 more days at 37 °C. Viral cytopathic effects (CPE) were observed under an inverted microscope and virus neutralization titers were determined as the reciprocal of the highest serum dilution that completely prevented the CPE.

Enzyme-linked immunosorbent assay (ELISA). The ELISA was performed using a recombinant receptor-binding domain (RBD) protein with a C-terminal HIS-tag purified by using TALON metal affinity resin from Expi293F cells (Thermo Fisher Scientific). The ELISA plates were coated overnight at 4 ºC with 50 µl of the RBD protein at a concentration of 2 µg/ml in phosphate-buffered saline (PBS). After being blocked with PBS containing 0.1% Tween 20 (PBS-T) and 3% milk powder, the plates were incubated in duplicate with heat-inactivated (56ºC for 30 minutes) serum diluted in PBS-T with 1% milk powder. After a 4-hour incubation at room temperature, the plates were washed with PBS-T three times and then incubated with a hamster IgG secondary antibody conjugated with horseradish peroxidase (Invitrogen; 1:7,000 dilution in PBS-T with 1% milk powder). After a 1-hour incubation with the secondary antibody, the plates were washed three times with PBS-T and then developed with SigmaFast o-phenylenediamine dihydrochloride solution (Sigma). After a 10-minute incubation, the reaction was stopped with the addition of 3 M hydrochloric acid. The absorbance was measured at a wavelength of 490 nm (OD_{490}). Background measurements from Day 0 plasma were subtracted from the Day 24 plasma for each dilution. The IgG antibody titer was defined as the highest plasma dilution with an OD_{490} cut-off value of >0.15.

Statistical analysis. Data are expressed as the mean ± SD. We used R (www.r-project.org) and lme4 (9) to perform a linear mixed effects analysis of the body weight and lung function data, which were normalized to the initial weight of each animal. As fixed effects, we used the different virus sample groups, and the time of measurement (with an interaction term between those fixed effects). As random effects, we had intercepts for the individual animals. We used the lsmeans (10) package to compare the groups at different timepoints, for each model separately, and the *p*-values were adjusted using Holm's method. To compare the titers of the different groups to each other, we used a one-way ANOVA followed by Tukey's post-hoc test or a two-tailed unpaired Wilcoxon's rank sum test with a continuity correction. *P* values of < 0.05 were considered statistically significant.

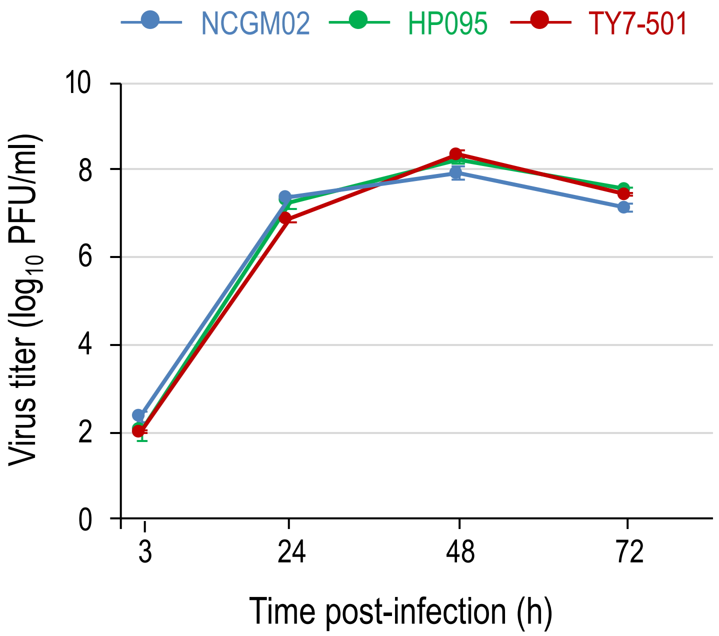

Fig. S1. Growth kinetics of SARS-CoV-2 viruses in cell culture.
VeroE6/TMPRSS2 cells were infected with viruses at an MOI of 0.001. The supernatants of the infected cells were harvested at the indicated times, and virus titers were determined by use of plaque assays on VeroE6/TMPRSS2 cells. Error bars indicate standard deviations from three independent experiments.

Table S1. Amino acid substitutions in the genomes of viruses used in this study compared to the reference genome of Wuhan/Hu-1/2019 (Wuhan-1)

	ORF1ab												
	1004	1176	188	1795	2702	2981	3557	3675	4715	5665	5968	7085	
Wuhan-1	T	A	S	K	Q	S	F	SGF	P	E	P	R	
NCGM02	T	V	S	K	Q	S	F	SGF	P	E	P	R	
HP095	T	A	S	K	H	F	F	SGF	L	E	L	I	
TY7-501	A	A	L	Q	Q	S	L	Del	P	D	P	R	
	Spike												
	18	20	26	138	181	190	417	484	501	614	655	1027	1176
Wuhan-1	L	T	P	D	G	R	K	E	N	D	H	T	V
NCGM02	L	T	P	D	G	R	K	E	N	D	H	T	V
HP095	L	T	P	D	G	R	K	E	N	G	H	T	V
TY7-501	F	N	S	Y	V*	S	T	K	Y	G	Y	I	F
	ORF3a		ORF8		N								
	95	253	84	92	80	203	204	234					
Wuhan-1	L	S	L	E	P	K	R	M					
NCGM02	L	S	S	E	P	R	G	M					
HP095	F	S	L	E	P	K	R	I					
TY7-501	L	P	L	K	R	K	R	M					

*This mutation (G181V) was not detected in the virus present in the clinical samples from the patient.
Gray shading indicates the receptor-binding domain of the spike protein

Table S2. ELISA endpoint titers of convalescent human sera or plasma against the RBD of NCGM02 or TY7-501*

Patient ID	Age	Onset day	Collection day	Days from onset	Sample type	RBD	
						NCGM02	TY7-501
HPCo-010	55	3/1/2020	6/3/2020	94	plasma	10240	5120
HPCo-015	49	3/16/2020	6/18/2020	94	plasma	10240	2560
HPCo-022	67	3/18/2020	6/25/2020	99	plasma	20480	10240
HPCo-025	52	4/1/2020	7/3/2020	93	plasma	10240	5120
HPCo-029	61	3/29/2020	6/29/2020	92	plasma	20480	5120
FSCo-001	75	2/8/2020	2/29/2020	21	serum	20480	10240
FSCo-002	67	2/3/2020	7/6/2020	154	serum	5120	5120
FSCo-003	80	2/14/2020	3/19/2020	34	serum	20480	10240
FSCo-004	64	3/29/2020	5/3/2020	35	serum	81920	40960
FSCo-005	62	4/1/2020	5/18/2020	47	serum	20480	10240
FSCo-006	77	5/23/2020	7/3/2020	41	serum	81920	40960
STCo-001	47	3/25/2020	4/10/2020	16	serum	20480	5120
STCo-002	38	3/23/2020	4/14/2020	22	serum	163840	40960
STCo-003	68	3/28/2020	4/20/2020	23	serum	81920	20480
STCo-005	59	4/3/2020	7/9/2020	97	plasma	10240	5120
STCo-006	38	4/24/2020	5/25/2020	31	plasma	40960	20480
STCo-007	64	4/4/2020	4/28/2020	24	serum	20480	10240
STCo-008	46	3/29/2020	6/3/2020	66	plasma	20480	10240
STCo-009	79	4/11/2020	7/7/2020	87	plasma	40960	20480
SUCo-001	57	3/5/2020	3/31/2020	33	plasma	163840	40960
SUCo-001	57	3/5/2020	5/20/2020	76	plasma	40960	20480
SUCo-002	60	3/21/2020	4/23/2020	26	plasma	327680	81920
SUCo-002	60	3/21/2020	5/7/2020	47	serum	81920	20480
SUCo-004	81	4/16/2020	6/1/2020	46	serum	327680	81920
SUCo-005	73	4/1/2020	5/28/2020	57	serum	81920	20480
EJCo-001	69	1/28/2020	2/18/2020	21	serum	655360	163840
EJCo-002	60	2/12/2020	2/25/2020	13	serum	20480	10240
EJCo-003	78	2/10/2020	2/25/2020	15	serum	81920	40960
EJCo-004	70	2/20/2020	3/2/2020	11	serum	40960	20480
HICo-002	64	2/3/2020	6/5/2020	123	plasma	40960	40960
HICo-002	64	2/3/2020	7/3/2020	151	plasma	40960	20480
HICo-004	67	2/24/2020	5/29/2020	95	plasma	655360	327680
KYCo-001	50	4/4/2020	7/9/2020	96	plasma	40960	10240
KYCo-002	65	4/9/2020	6/4/2020	56	plasma	163840	20480
NCCo-503	48	1/2/2021	1/22/2021	20	serum	20480	40960

*Viral antibody endpoint titers against the receptor-binding domain (RBD) expressed as the reciprocal of the highest dilution with an OD_{490} cutoff value >0.15.

Table S3. ELISA endpoint titers of BNT162b2-vaccinated human sera against the RBD of NCGM02 or TY7-501*

Vaccinated human sera	Sample ID	RBD	
		NCGM02	TY7-501
15–21 days after the first immunization	1785	1280	1280
	2946	2560	1280
	9163	1280	1280
	2955	640	320
	8787	640	640
	8832	1280	640
	11182	1280	1280
7–14 days after the second immunization	7482	40960	10240
	11462	40960	10240
	1610	10240	10240
	4500	40960	20480
	1497	20480	10240
	4719	10240	10240
	4704	40960	20480
	7635	20480	20480
	7533	20480	10240
	4608	20480	20480
	7629	10240	5120
	7854	20480	10240
	11471	10240	10240
15–20 days after the second immunization	7806	20480	10240
	7644	10240	5120
	7455	5120	5120
	4605	10240	5120
	7683	10240	5120
	7602	10240	5120
	9418	10240	5120
At least 21 days after the second immunization	7656	20480	10240
	7614	10240	2560
	7794	40960	10240
	9394	40960	5120
	7626	10240	5120
	7686	10240	5120
	7641	5120	2560
	7701	20480	5120
	7836	20480	10240

*Viral antibody endpoint titers against the receptor-binding domain (RBD) expressed as the reciprocal of the highest dilution with an OD_{490} cutoff value >0.15.

Table S4. Primary infection with HP095 protects hamsters from re-challenge with TY7-501*

Primary infection	Animal ID	Neutralizing antibody titer in serum[†]	Virus titers (log_{10} PFU/g) of animals infected with HP095		Virus titers (log_{10} PFU/g) of animals infected with TY7-501	
			Nasal turbinate	Lung	Nasal turbinate	Lung
Mock (PBS)	#1	<10	8.5	8.6	NA[‡]	NA
	#2	<10	8.4	8.8	NA	NA
	#3	<10	8.4	8.7	NA	NA
	#4	<10	7.6	8.7	NA	NA
HP095	#5	1280	—[§]	—	NA	NA
	#6	640	6.4	—	NA	NA
	#7	1280	—	—	NA	NA
Mock (PBS)	#8	<10	NA	NA	8.4	8.4
	#9	<10	NA	NA	8.2	8.4
	#10	<10	NA	NA	8.3	8.2
	#11	<10	NA	NA	8.2	8.1
HP095	#12	320	NA	NA	6.4	—
	#13	640	NA	NA	—	—
	#14	640	NA	NA	1.9	—
	#15	640	NA	NA	2.6	—

*Syrian hamsters were intranasally inoculated with 103 PFU (in 30 µl) of SARS-CoV-2/UT-HP095-1N/Human/2020/Tokyo (HP095) or hCoV-19/Japan/TY7-501/2021 (TY7-501) on Day 21 after primary infection with HP095. Three or four Syrian hamsters per group were euthanized on Day 3 after re-challenge for virus titration.

[†]Viral neutralization titers against HP095; sera were collected on Day 20 after the primary infection.

[‡]NA, not applicable.

[§]—, virus not detected. The detection limit was 10 PFU/g.

Table S5. Primary infection with NCGM02 protects hamsters from re-challenge with TY7-501*

Primary infection		Animal ID	Antibody endpoint titer in serum†	Neutralizing antibody titer in serum‡	Day of organ collection post-infection	Virus titers (log_{10} PFU/g) of animals infected with NCGM02		Virus titers (log_{10} PFU/g) of animals infected with TY7-501	
Virus	Infection dose					Nasal turbinate	Lung	Nasal turbinate	Lung
NCGM02	10^3 PFU	#22	20480	320	Day 3	2.3	—§	NA¶	NA
	10^3 PFU	#24	20480	320		—	—	NA	NA
	10^2 PFU	#16	20480	160		—	—	NA	NA
	1 PFU	#9	10240	160	Day 6	—	—	NA	NA
	10 PFU	#11	10240	160		—	—	NA	NA
	10 PFU	#13	10240	160		—	—	NA	NA
	10^3 PFU	#20	20480	320	Day 3	NA	NA	4.7	—
	10^3 PFU	#21	20480	320		NA	NA	3.6	—
	10^2 PFU	#15	20480	320		NA	NA	3.0	—
	10 PFU	#14	10240	160	Day 6	NA	NA	—	—
	10^2 PFU	#17	10240	320		NA	NA	—	—
	10^2 PFU	#18	10240	160		NA	NA	—	—

*Syrian hamsters were intranasally inoculated with 10^3 PFU (in 30 μl) of SARS-CoV-2/UT-NCGM02/Human/2020/Tokyo (NCGM02) or hCoV-19/Japan/TY7-501/2021 (TY7-501) 9 months after primary infection with NCGM02. Three Syrian hamsters per group were euthanized on Days 3 and 6 after re-challenge for virus titration.

†Viral antibody endpoint titers against the receptor-binding domain expressed as the reciprocal of the highest dilution with an OD_{490} cutoff value >0.15; sera were collected 9 months after the primary infection.

‡Viral neutralization titers against NCGM02; sera were collected 9 months after the primary infection.

§—, virus not detected. The detection limit was 10 PFU/g.

¶NA, not applicable.

SI References

1. S. Matsuyama *et al.*, Enhanced isolation of SARS-CoV-2 by TMPRSS2-expressing cells. *Proc Natl Acad Sci U S A* **117**, 7001-7003 (2020).
2. M. Imai *et al.*, Syrian hamsters as a small animal model for SARS-CoV-2 infection and countermeasure development. *Proc Natl Acad Sci U S A* **117**, 16587-16595 (2020).
3. K. M. Braun *et al.*, Transmission of SARS-CoV-2 in domestic cats imposes a narrow bottleneck. *PLoS Pathog* **17**, e1009373 (2021).
4. J. Quick, nCoV-2019 sequencing protocol. *protocolsio* (2020).
5. E. W. Lamirande *et al.*, A live attenuated severe acute respiratory syndrome coronavirus is immunogenic and efficacious in golden Syrian hamsters. *J Virol* **82**, 7721-7724 (2008).
6. A. Roberts *et al.*, Severe acute respiratory syndrome coronavirus infection of golden Syrian hamsters. *J Virol* **79**, 503-511 (2005).
7. A. Roberts *et al.*, Therapy with a severe acute respiratory syndrome-associated coronavirus-neutralizing human monoclonal antibody reduces disease severity and viral burden in golden Syrian hamsters. *J Infect Dis* **193**, 685-692 (2006).
8. M. Chung *et al.*, CT Imaging Features of 2019 Novel Coronavirus (2019-nCoV). *Radiology* **295**, 202-207 (2020).
9. D. Bates, Mächler, M, Bolker, B, Walker, S, "Fitting Linear Mixed-Effects Models Using lme4." *J Stat Softw* **67**, 1–48 (2015).
10. R. V. Lenth, Least-Squares Means: The R Package lsmeans. *J Stat Softw* **69**, 1-33 (2016).
